# Spectral Decomposition of the Flow and Characterization of the Sound Signals through Stenoses with Different Levels of Severity

**DOI:** 10.3390/bioengineering8030041

**Published:** 2021-03-19

**Authors:** Fardin Khalili, Peshala T. Gamage, Amirtahà Taebi, Mark E. Johnson, Randal B. Roberts, John Mitchell

**Affiliations:** 1 Department of Mechanical Engineering, Embry-Riddle Aeronautical University, 1 Aerospace Boulevard, Daytona Beach, FL 32114, USA; 2 Department of Biomedical and Chemical Engineering and Sciences, Florida Institute of Technology, 2930 Science Cir., Melbourne, FL 32901, USA; pthibbotuwawagam@fit.edu; 3 Department of Biomedical Engineering, University of California Davis, One Shields Avenue, Davis, CA 95616, USA; ataebi@ucdavis.edu; 4 Telecraft Engineering Inc., 1254 Mount Carmel Church Lane, Canton, GA 30114, USA; mjohnson@telecraft.com (M.E.J.); randy.roberts@telecraft.com (R.B.R.); 5 Infrasonix Inc., 3100 Breckinridge Blvd, Unit 712, Duluth, GA 30096, USA

**Keywords:** atherosclerosis, stenosis, proper orthogonal decomposition, turbulent pressure fluctuations, sound source localization, break frequency, frequency spectral analysis

## Abstract

Treatments of atherosclerosis depend on the severity of the disease at the diagnosis time. Non-invasive diagnosis techniques, capable of detecting stenosis at early stages, are essential to reduce associated costs and mortality rates. We used computational fluid dynamics and acoustics analysis to extensively investigate the sound sources arising from high-turbulent fluctuating flow through stenosis. The frequency spectral analysis and proper orthogonal decomposition unveiled the frequency contents of the fluctuations for different severities and decomposed the flow into several frequency bandwidths. Results showed that high-intensity turbulent pressure fluctuations appeared inside the stenosis for severities above 70%, concentrated at plaque surface, and immediately in the post-stenotic region. Analysis of these fluctuations with the progression of the stenosis indicated that (a) there was a distinct break frequency for each severity level, ranging from 40 to 230 Hz, (b) acoustic spatial-frequency maps demonstrated the variation of the frequency content with respect to the distance from the stenosis, and (c) high-energy, high-frequency fluctuations existed inside the stenosis only for severe cases. This information can be essential for predicting the severity level of progressive stenosis, comprehending the nature of the sound sources, and determining the location of the stenosis with respect to the point of measurements.

## 1. Introduction

The build-up of fatty material and calcium inside an artery can, over time, form a plaque at the lumen surface, narrowing the lumen area and subsequently limiting the blood flow to vital organs of the body. This adverse condition is known as atherosclerosis, a chronic, progressive inflammatory disease with a long asymptomatic phase [1]. Atherosclerosis is mostly associated with coronary artery disease (CAD), limiting the oxygen-rich blood to the myocardium. It is known as the leading cause of death worldwide [2]. Not only about 18.2 million people are affected by CAD in the United States alone [3], but also CAD is a severe disorder responsible for most sudden deaths of adults over the age of 20 [4]. Atherosclerosis lesions can also develop in carotid and peripheral arteries, leading to stroke and peripheral artery occlusive disease (PAOD), respectively. PAOD can lead to severe complications such as critical limb ischemia.

The asymptomatic nature of atherosclerosis limits the early diagnosis of the disease, leading to more deaths. Hence, early-stage identification of atherosclerosis through non-invasive predictive tools is vital to avoid these complications. If detected early, treatments can be adapted to prevent artery rupture and improve the patient’s condition. An invasive procedure of arteriography (or angiography) is the most common method for diagnosing stenosis, usually employed after detecting clinical symptoms. In this process, an X-ray image is obtained after injecting X-ray contrast agents into the bloodstream. Although widely used, the invasive nature of arteriography requires placement of a catheter at the site of a stenosed artery, which can cause complications such as bleeding and infections. This procedure may also underestimate the stenosis severity, as it evaluates a projected 2D view of the artery [5]. Non-invasive alternatives to arteriography, such as Doppler ultrasonography, computed tomographic angiography, and magnetic resonance imaging, are available, yet these technologies can be expensive and time-consuming. Hence, low-cost, non-invasive, easily accessible methods that can effectively diagnose and monitor atherosclerosis progression from early stages can help save lives and reduce the associated cost. In this context, the analysis of acoustics and vibration of a human body (caused by the presence of stenosis) may pave the way to non-invasive diagnostic methods as developed for many fields in biomechanics [6,7].

Stenosis inside an artery can alter the laminar blood flow behavior to turbulence due to the high velocity generated at the constriction. This will lead to high-frequency turbulent pressure fluctuations downstream of the stenosis generating vascular sounds, often referred to as murmurs [8,9]. Subsequently, the induced pressure fluctuations can transmit through the surrounding soft tissues to the epidermal surface [10,11]. In 1970, Lees and Dewey proposed the diagnosis technique, Phonocardiography, which utilized vibro-acoustics on the epidermal surface caused by the flow fluctuations inside an artery in the presence of stenosis [12]. Their study showed similarities between the sound spectra obtained from in vivo measurements and laboratory investigations of turbulent pipe flow, suggesting that the analysis of acoustic signals on the epidermal surface can provide critical clinical information for atherosclerosis diagnosis. Another study [13] investigated the flow dynamics in a realistic stenosed vessel and observed high-frequency velocity fluctuations within the audible range of 100–300 Hz, generated by the vortex shedding downstream of the stenosis. The experimental and numerical studies by Salman and Yazicioglu reported significant acoustic radiation in the range of 250 to 600 Hz for stenosis severities over 70% [14,15]. The experimental study by Tobin and Chang [16] analyzed the flow-induced sound generation for various flow velocities and stenosis severities. Their study reported parameters to find an empirical relation to estimating the turbulent pressure field for constricted flows. Another experimental study by Borisyuk [17] suggested that 50% of lumen area reduction can result in a tenfold increase in radiated acoustic power. Mittal et al. numerically studied acoustics in a planar channel with a one-sided semi-circular constriction subjected to pulsatile flow. They concluded that there are direct relations between arterial murmurs and arterial pressure fluctuations [9]. In another study, the acoustic radiation analysis in the 2D vessel model with 50% and 75% severities showed a significant increase in radiated acoustic power with an increase in severity [18]. Khalili et al. [11] performed a similar numerical study employing different numerical methods and reported comparable results.

The study of frequency contents of flow structures in unhealthy arteries has recently been an active research area in biomechanics. In some similar studies done by the authors of this paper, the wall pressure spectra measured downstream of the stenosis were analyzed using spectral decomposition techniques [11,19,20]. These studies explored the coherent flow structures related to the peak frequencies. Bakhshinejad et al. [21] used proper orthogonal decomposition (POD) and a de-noising method to reconstruct flow structures, collected by 4D-Flow MRI, through a cerebral aneurysm. They concluded that the POD method was more suitable for preserving and visualizing the small flow structures than the other method. In another study, Janiga [22] extracted the blood flow features using POD to illustrate the two most energetic modes of the flow associated with the mean temporal velocity and the secondary flow. The POD analysis was also conducted for stenosed carotid arteries to identify and quantitatively measure the instabilities of the transitional and turbulent flows [23,24]. Natarajan et al. [25] also used a temporal filtering methodology to decompose the flow velocity at different frequency bandwidths and map the filtered flow spatially within the domain. The use of POD and flow decomposition was also described in prior study for a highly disturbed flow through a stenosis [26].

It is evident that the investigations on the correlation of stenosis severity and the generated acoustics can help enhance our understanding of the genesis of sound sources and potentially relate them to the flow structures associated with different disease conditions. The current study focused on a detailed analysis of the flow variations caused by progressive stenosis. The objective was to identify the acoustic sources in the flow responsible for the high-frequency fluctuations potentially captured on the epidermal surface. This was achieved with the use of high-fidelity computational fluid dynamics (CFD) to accurately capture turbulence in a stenosed artery at different severity levels of 0%, 20%, 40%, 50%, 70%, 87%, and 92%, combined with state-of-the-art sound analysis techniques, such as frequency spectral analysis, POD, and a frequency-based temporal filtering method, to characterize the acoustic signals generated with the progression of the stenosis. The paper is structured as follows: (a) CFD simulations were validated against experimental measurements; (b) the flow domain was investigated to focus on the variation of the mean axial velocity in the post-stenotic region, flow fluctuations within the artery, and root-mean-square (RMS) of pressure fluctuations on the artery wall; (c) the point of maximum excitation, due to flow fluctuations, was localized inside the artery and on the artery wall; (d) frequency spectral analysis was performed to analyze the flow-generated acoustics in the spectral domain and to provide an acoustic spatial-frequency map of the post-stenotic region; (e) the flow domain was spectrally decomposed to identify the location of the acoustic sources in specific frequency bandwidths. Moreover, sound characteristics, such as break frequency, were suggested as the predictive indicators of stenosis severity at different stages. The proposed methodology of this study can help derive sound features from predicting the progression of atherosclerosis by analyzing epidermal vibrations caused by stenosed arteries.

## 2. Materials and Methods

### 2.1. Computational Model

**Figure 1:**
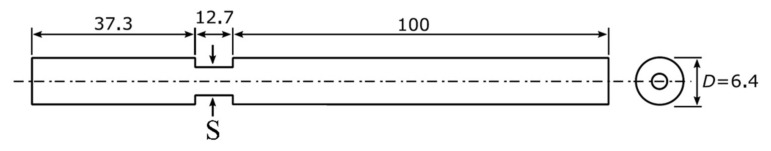
A sectional view of the flow domain. Flow is from left to right. The figure is out of scale, and the dimensions are in mm.

The complexity of the exact shape of arterial stenosis varies among patients, and it is challenging to model it accurately [27]. Hence, a simplified model of stenosis was considered in similar investigations [14,28,29], as well as in the current study. The schematic of the flow domain is shown in Figure 1. The severity of the stenosis depends on the reduction in flow area, calculated by (1),

(1) $$S=\;\left(\frac{D^{2}-d^{2}}{D^{2}}\right)\times 100\%$$

where *S* denotes severity of stenosis, *D* = 6.40 mm is the vessel diameter, and *d* is the diameter at the stenosis equal to 1.18 mm, 2.31 mm, 3.51 mm, 4.53 mm, 4.96 mm, 5.72 mm, 6.4 mm, for 92%, 87%, 70%, 50%, 40%, 20%, and 0% stenosis, respectively.

The current model dimensions were selected based on the measurements of peripheral arteries in previous studies [30,31]. Due to the turbulent nature of the flow, the simulations were performed as three-dimensional.

### 2.2. Physics and Flow Conditions

According to the diameter of peripheral arteries, the blood flow velocity can vary from 0.066 to 0.642 m/s [32], corresponding to Reynolds numbers ranging from 100 to 2200. In current simulations, the velocity of 0.3125 m/s (equivalent to Re = 2000) was specified at the inlet to represent a relevant critical flow condition [14,16,33]. A higher Reynolds number leads to higher fluctuations and stronger sound signals, which is desirable when analyzing the frequency content of a highly disturbed flow. A sufficient entrance length of 37.3 mm was considered to ensure that the inflow was fully developed before the stenosis. The flow was also considered steady since the cardiac cycle frequency is in the order of 1 Hz, in contrast to the frequencies of 20–1000 Hz in the post-stenotic region. Density and kinematic viscosity of water, as a typical blood substitute used in in-vitro experiments, were set to 1000 kg/m^3^ and 10^−6^ Pa.s, respectively. Reference pressure was zero at the outlet, and the no-slip boundary condition was used at the wall boundaries.

Numerical analysis was conducted using Simcenter STAR-CCM+ (2020.1.1, CD-Adapco, Siemens PLM, Plano, TX, USA). The dynamic Smagorinsky turbulence model was applied to simulate transitional flow with appropriate scale resolving simulation (SRS) modeling. This model was suggested in the literature to simulate flows inside stenotic vessels [34,35]. The study of time-independence determined that a time-step of 2.5 × 10^−5^ s was adequate for accurate solution of the turbulent transients and keep the Courant number close to 1. In addition, time step convergence of less than 10^−4^ was achieved, which is particularly important with SRS models [36]. The governing equations were also discretized using second-order central discretization in space and second-order implicit discretization in time to deliver accurate results.

### 2.3. Proper Orthogonal Decomposition (POD) Analysis

For the post-processing of the results obtained from the CFD analysis, the coherent flow structures through the stenosis were reconstructed using the POD method to visualize the sound sources through the stenosis. The POD method decomposes the time-varying flow field into spatial and temporal parts, describing the modes that represent the coherent flow structures in the flow and the time evolution of these modes, respectively. Hence, the POD and frequency-based temporal filtering of the flow can provide insightful information of the acoustic sources in arteries valuable to clinicians. For example, in previous studies, it was concluded that the spatial resolution of 4D-Flow MRI is not sufficient for small vessels to resolve flow features concisely [37], while POD can reconstruct features of complex recirculating flows [38]. In terms of its application feasibility, it should be noted that the POD technique has also been used for the flow through mechanical aortic valve [39], cerebral aneurysm [40,41], and coronary arteries [42].

In this study, the pressure flow field was selected for POD analysis to provide useful information on the localization of sound sources with maximum pressure fluctuations within the stenosis and post-stenotic region. POD can be formulated as,

(2) $$p\left(X,t\right)≅\overset{M-1}{\underset{i=0}{\sum }}\mu_{i}\left(t\right)\varphi_{i}\left(X\right)$$

where a time-varying quantity, $p\left(X,t\right)$, is represented as a summation of linearly independent (i.e., orthogonal) mode shapes $\varphi_{i}\left(X\right)$ multiplied by their time-varying amplitudes $\mu_{i}\left(t\right)$. These modes are ordered according to their energy content (i.e., the most energetic mode first). When M → ∞, the summation of mode shapes perfectly represents $p\left(X,t\right)$.

Following procedure was used to calculate the POD modes, $\varphi_{i}(X$), and time evolvements, $\mu_{i}\left(t\right)$, from the pressure field data in the fluid domain:

Step1: Create a snapshot matrix U, which contains the pressure data $p\left(X,t\right)$. Here, U is n×m  matrix, where n is the number of nodal points in the CFD mesh and m is the number of time steps.

Step2: Find V by performing singular value decomposition (SVD) on snapshot matrix U,

(3) $$U=VDW^{T}$$

As denoted in (3), SVD finds V and W which are orthonormal matrices, and the diagonal matrix D which contains the singular values. These singular values represent the energy percentage of each mode. Columns of matrix V contain the POD modes $\varphi_{i}(X$) of the system arranged from the highest energy mode to the lowest energy modes in descending order as shown in (4). The highest energy mode represents the average flow quantity, often referred to the 0th mode.

(4) $$V=\varphi_{i}=\left[\varphi_{0}\left(X\right)\ldots \ldots \varphi_{i}\left(X\right)\ldots \ldots \ldots \varphi_{M-1}\left(X\right)\right]$$

Then, the time evolvement $\mu_{i}\left(t\right)$ of respective mode $\varphi_{i}(X$) is calculated using (5), based on the orthogonality condition of $\varphi_{i}(X$). The spectrum of time evolvement $\mu_{i}\left(t\right)$ of each mode delivers the frequency information of the respective mode.

(5) $$\mu_{i}\left(t\right)=\left[\begin{array}{ll} \mu_{i}\left(t_{0}\right) & \begin{array}{cc} \mu_{i}\left(t_{1}\right) & \begin{array}{cc} \ldots \ldots \ldots \ldots & \mu_{i}\left(t_{m}\right) \end{array} \end{array} \end{array}\right]=\varphi_{i}{}^{T}U_{m}$$

### 2.4. Fast Fourier Transform (FFT)

The time history of pressure fluctuations was recorded at 41 nodes on the vessel wall in the post-stenotic region, each separated 2.5 mm apart, for the last 1.92 s of the flow solution at a sampling frequency of 4 kHz. This sampling frequency was sufficient to show the frequency content of the flow up to 2 kHz. The data were then post-processed using MATLAB to transform into a spectral domain by performing Hanning window filtering and fast Fourier transform (FFT) computation. The energy of pressure fluctuations was then converted to a logarithmic scale,

(6) $$p\left(\mathrm{dB}\right)=20\log_{10}\left(\frac{P}{p_{ref}}\right)$$

where P is the computed pressure fluctuations on the wall in pascals, $p\left(dB\right)$ is the sound pressure level converted into dB, and $p_{ref}$ is the reference pressure set to 1 Pa, similar to previous studies [16,29].

Furthermore, to find the origins of these frequencies, spectral filtering of the flow data was adapted. To visualize the spectral decomposed acoustic pressures, we applied temporal filtering to the pressure fluctuations, $p^{\prime }$. Here, time series of $p^{\prime }$ values at each mesh node were forward–backward filtered through a bandpass 6th order Butterworth filter with specified cutoff frequencies. These filters were designed using MATLAB signal processing toolbox (2020b. The MathWorks, Inc., Natick, MA, USA).

### 2.5. Mesh

The use of scale resolving simulation (SRS) turbulence models for wall-bounded flows requires high-quality mesh while it is finer in those areas where high physical gradients are present; it is also essential to maintain y^+^ ≤ 1 [43,44]. To increase the accuracy of the flow solution, a solution-based mesh refinement was conducted based on turbulent kinetic energy (TKE) as an indication of flow fluctuations and the energy of sound sources. This was accomplished through the following steps: (a) generate an initial coarse mesh on the geometry; (b) solve a steady-state flow and use TKE to threshold and flag the cells that require refining; (c) create a field function to specify the new cell size for the flagged cells for refinement; (d) create a refinement table for the entire domain with the refinement field function as the scalar, and extract the values; (e) add the refinement table to your volume mesher and re-generate the volume mesh. This way, the mesh was optimally refined based on an important flow parameter in the region of interest to avoid unnecessary mesh cells throughout the domain and reduce computational costs. This semi-automated method reduced computational times by about 30% compared to manual meshing based on different regions (inlet—stenosis—fluctuating zone—reattachment and laminar flow regions). The mesh was determined suitable for the large-eddy simulation (LES) simulation after calculating the ratio of cell size to the minimum Kolmogorov length scale obtained in the fluctuating region (1D to 4D downstream of stenosis). The maximum cell size in this region was set to 0.144 mm to attain the ratio of below 20, which is within the maximum allowable range suggested by [45].

In addition, a grid-independent study was conducted to find the optimized mesh configuration. Four different mesh configurations were set up with approximately 400 k, 700 k, 1.4 M, and 2 M mesh cells while keeping the Courant number close to 1 and y^+^ ≤ 1. The evaluation of the mean velocity in flow direction along the pipe indicated that mesh 3 with 1.4 M mesh cells was the most appropriate mesh configuration. In addition, an accurate prediction of pressure drop in the flows with separation depends on resolving the velocity gradients normal to the wall. Hence prism layers were chosen as they allow the solver to resolve near-wall flow more accurately. A 10-layer prism layer mesh with a total thickness of 0.059 mm and layer stretching factor of 1.35 was employed near the boundaries to resolve the velocity gradients normal to the wall.

### 2.6. Validation

**Figure 2:**
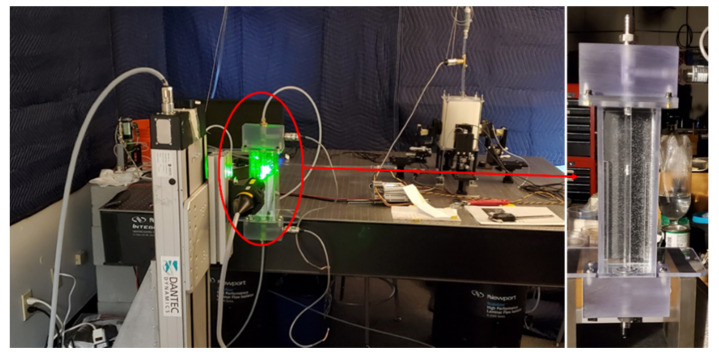
Experimental setup of Laser Doppler Anemometry (LDA) axial velocity measurements for a constricted pipe representing arterial stenosis.

In the current study, Laser Doppler Anemometry (LDA) (Dantec Dynamics A/S., Skovulunde, Denmark), as a non-intrusive method that does not interfere with the flow field and sound generation, was used to measure the velocity at different locations in the upstream and post-stenotic regions to validate numerical results. Figure 2 displays the experimental setup for a constricted pipe (a simplified model of a stenosed artery). The velocity measurements using an LDA system were performed for 30 seconds at each location and sampling frequencies of more than 1000 Hz.

**Figure 3:**
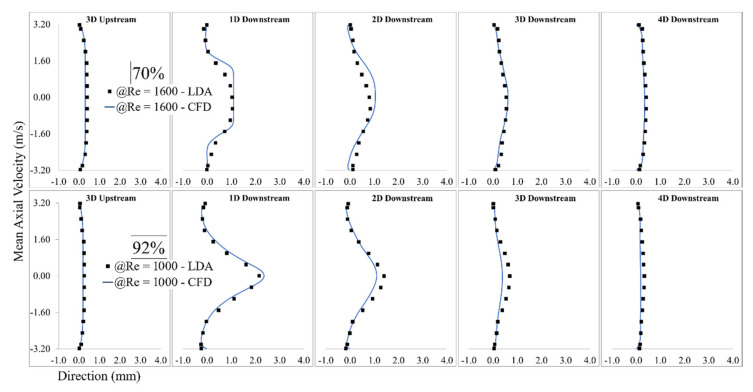
Validation of computational fluid dynamics (CFD) results of 92% stenosis at Re = 1600 with the LDA measurements.

LDA measurements were performed at different locations downstream of the constricted region (from 3D upstream to 8D downstream), each separated by one diameter. The results of one location upstream of the stenosis and four locations in the post-stenotic region with the highest intensity of pressure fluctuations and instability in the flow are shown in Figure 3. The validation was done for several severity levels and Reynolds numbers such as 70% at Re = 1600 and 92% at Re = 1000. Note that the correlation of sound sources with the progression of stenosis severity at different Reynolds numbers and for different types of stenosis is the objective of another study that we will conduct in future works after the current analysis.

**Figure 4:**
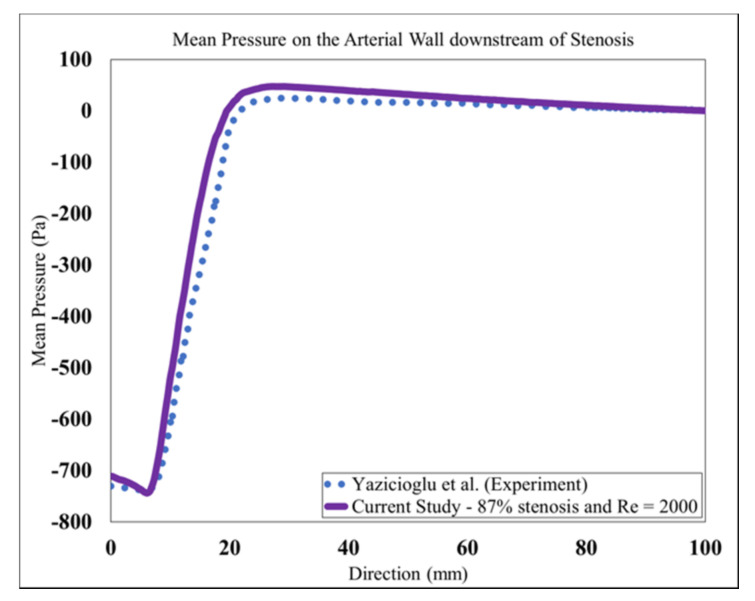
Mean Pressure on the arterial wall in the post-stenotic region.

Mean wall pressure along the artery wall in the post-stenotic region was also selected for additional mesh-independence study. The mean pressure at the wall along the artery is shown in Figure 4. The results were validated with respect to a previous experimental study [29]. In that experimental study, a catheter-type pressure transducer was used to collect mean and fluctuating pressure signals within the vessel lumen. The mean wall pressure in the current study showed a good agreement for both wall pressure distribution and the location of the maximum pressure. As blood flow entered the expansion region, pressure distribution changed significantly, leading to a wall pressure difference of about 700 Pa between the stenosis and flow domain exits. Additionally, mean wall pressure reached its maximum value at about 25 mm (or x = 4D), after which it decreased gradually toward the exit.

The validation results showed that the LES turbulence model had a good agreement with experimental results, especially in the regions where the flow separation occurs, and flow experiences the highest fluctuation about 1D to 4D downstream of the stenosis.

## 3. Results

The flow was initially solved for about 2.4 s to ensure that the mean flow reached steady state (i.e., five times 0.48 s, which was the time required for a fluid element to travel from inlet to outlet). Thus, the transient effects on the mean flow parameters were diminished before recording all flow parameters. The flow solution, including mean velocity components and the pressure, was then obtained by averaging these flow fields for the last 1.92 s of simulation (total physical simulation time of 4.32 s) to deliver accurate results.

**Figure 5:**
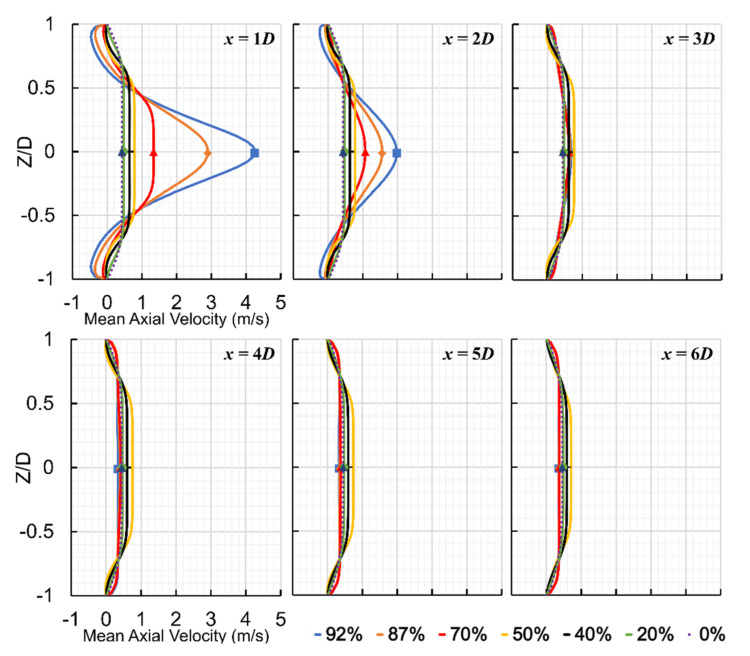
Mean axial velocity at six different locations (1D to 6D) downstream of stenosis for all severity cases.

The axial velocity significantly increased at the beginning of the stenosis due to a flow area reduction. It formed a flow jet at the exit of the stenosis and in the recirculation region, leading to a severe pressure drop. The maximum velocity in the flow direction increased to about 5.67 m/s, 3.60 m/s, 1.53 m/s, 0.79 m/s, 0.65 m/s, 0.52 m/s, 0.43 m/s (fully developed flow) for 92%, 87%, 70%, 50%, 40%, 20%, and 0% severities, respectively. The maximum axial velocity was about 18, 12, and 5 times larger than the inflow velocity for the most severe cases (i.e., 92%, 87%, and 70%, respectively). Figure 5 shows the mean axial velocity for all severity cases at six different locations (1D to 6D) downstream of the stenosis. As the flow jet entered the expansion post-stenotic region, the 92% stenosis led to the highest maximum mean axial velocity at 1D, followed by 87% and 70% cases. On the other hand, mean axial flow velocity was observed with small differences in the least severe cases (i.e., 50%, 40%, and 20%) than the healthy case (0%), mostly close to the wall right after the stenosis in the recirculation region, indicating slight disturbances to the flow. However, the slightly disturbed flow with small fluctuations was extended further downstream of the stenosis up to x = 6D. For these least severe cases, mean axial velocity and flow fluctuations remained low throughout the stenosis and post-stenotic region.

For the most severe cases, at 1D downstream of the stenosis, the shear layers around the flow jet became unstable, breaking into smaller eddies. The region between 1D and 4D downstream of the stenosis contained the highest flow fluctuations. It was noticed that as the mean axial velocity decreased, the flow fluctuations increased, representing a fluctuating zone with the highest sound sources. Around x = 4D, vortical structures started to lose their strength, and whereafter, fully developed turbulent flow was observed in the reattachment and stabilized zones.

**Figure 6:**
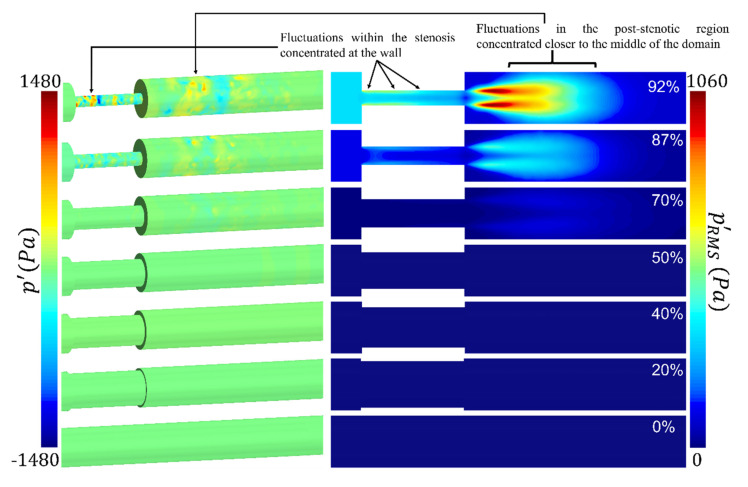
Pressure fluctuations on the arterial wall and root-mean-square (RMS) of pressure fluctuations on the middle cross-section of the flow domain showing the concentration and high-energy fluctuation through and downstream of the stenosis.

Acoustic pressure fluctuations, known as cardiovascular sounds, captured with a stethoscope, are correlated with the vibration of the epidermal surface as a result of propagated sound waves from the vessel through the tissue. Pressure fluctuations, especially on the internal arterial wall, are the focus of this study. They are accounted for as the primary sources of sound due to the structural response of the surrounding tissue. To get an overview of the flow solution, instantaneous pressure fluctuations ($p^{\prime }$) on the arterial wall and root-mean-square (RMS) of pressure fluctuations ($p_{RMS}^{\prime }$) at the middle cross-section of the flow domain are shown in Figure 6. Although low-pressure fluctuations existed in severity levels of 20%, 40%, and 50%, they are not visible in this figure due to the pressure fluctuation contours plotted for the same range for all cases, intended for comparison purposes. It was found that, as the severity of stenosis increased, the flow fluctuations appeared within the stenosis close to the wall. It then spread into the expansion region, concentrated in the fluctuating zone (from 1D to 4D downstream of the stenosis). Wall pressure fluctuations express the potential transmission of arterial wall vibrations to the epidermal surface. There was almost no sign of strong excitation on the arterial wall throughout the domain in the healthy and the least severe cases.

For the 70% stenosis, wall pressure fluctuations were observed in the post-stenotic region while the flow jet remained slightly unstable inside the stenosis. For 87% and 92% of cases, due to the length of the stenosis and a significant reduction in the flow area, the flow jet became significantly unstable inside the stenosis, turning to a highly turbulent flow before flow reaches the exit of the stenosis. From a fluid dynamics perspective, this can be considered as a method of diagnosis of severe cases. These pressure fluctuations inside the stenosis (displayed by $p_{RMS}^{\prime }$ in Figure 6) were more concentrated at the wall (i.e., plaque surface) compared to the fluctuations in the post-stenotic region, which were concentrated at the shear layer of the flow jet. The high concentrated pressure fluctuations, with the presence of turbulence, combined with high wall shear stresses at the plaque surface, can cause a fracture in atheromatous plaques, leading to embolism and stroke. However, depending on the stiffness of the plaque’s material (which may include calcifications) and the mechanical coupling of the artery to the body (which can induce vibrational modes), the effect of these high-concentrated fluctuations at the plaque surface can attenuate or amplify. It was noted that the study of wall pressure fluctuations and sound sources inside the stenosis is rare in the literature. First, this suggested us to examine this phenomenon in this study briefly, and secondly, it motivated us for further investigations to find the correlation between different variables such as the length of the stenosis and wall pressure fluctuation in future studies.

The RMS of pressure fluctuations in the middle cross-section showed that the energy of the pressure fluctuations was the highest through the stenosis, close to the wall, and 1D to 4D downstream of the stenosis, for 92% severity. It was also observed that the energy of pressure fluctuations in the post stenotic region elevated with an increase in the severity level beyond 70%. After 4D downstream of the stenosis, the energy of turbulent pressure fluctuations started to dissipate, at which eddies further broke into smaller ones. It is essential to fully understand the flow structure through the stenosis and accurately estimate the highly turbulent fluctuating region. During coronary catheterization measurements such as fractional flow reserve (FFR), it is required to move the probe further downstream (here, x > 4D) of the stenosis to avoid the turbulence region for accurate measurements.

**Figure 7:**
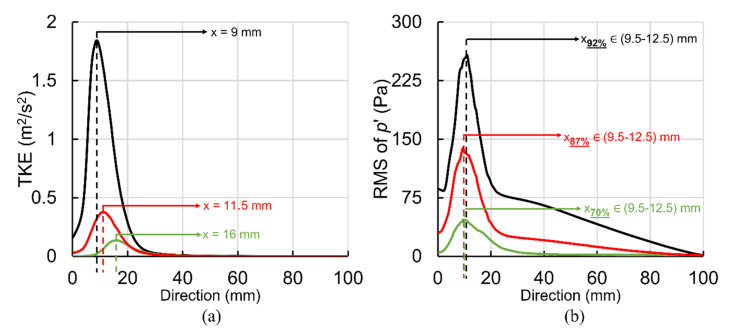
Point of maximum excitation in the post-stenotic region by analyzing (**a**) RMS of pressure fluctuations on the wall and (**b**) turbulent kinetic energy (TKE) on the stenosis centerline.

For sound source localization, it is necessary to find the point of maximum excitation in the post-stenotic region. Figure 7 shows the variation of turbulent kinetic energy (TKE) along the centerline of the stenosis and RMS of pressure fluctuations along the wall for the most severe cases. The TKE and $p_{RMS}^{\prime }$ values at lower severities below 50% were insignificant compared to the values observed in the most severe cases. It can be seen in Figure 7a, the point of maximum TKE was localized at about 1.5D, 2D, and 2.5D downstream of stenosis for 92%, 87%, and 70%, respectively. The TKE increased exponentially as the severity level increased. The TKE increased from the exit of the stenosis reaching the highest level before it was reduced rapidly as the flow became reattached and laminarized (after x = 4D). It was also noted that the point of maximum TKE moved towards the stenosis with the increase in severity. In contrast to the TKE, the point of maximum acoustic pressure on the wall was found in the same region, as seen in Figure 7b. This can be due to the length of the recirculation region determined to be around 2D for all these cases. Similar results were concluded in a previous experimental study [16]. With the existence of flow recirculation, the RMS of pressure fluctuations started at around 86 Pa, 30 Pa, and 5 Pa at the exit of stenosis on the wall and increased to maximum values of about 258 Pa, 139 Pa, and 47 Pa (for 92%, 87%, and 70%, respectively) in the region of 9.5 to 12.5 mm downstream of stenosis (or about x = 2D). These results suggested that the energy of the pressure fluctuations (potentially seen on the epidermal surface) can be one of the main features of stenosis severity, which can also be adapted to monitor a stenosis progression. Here, we can conclude that the energy levels of acoustic measurements using an attached microphone on the skin surface over the site of the stenosed artery and during induced hyperemia (i.e., during high flowrate) can serve as a potential indicator of stenosis severity.

**Figure 8:**
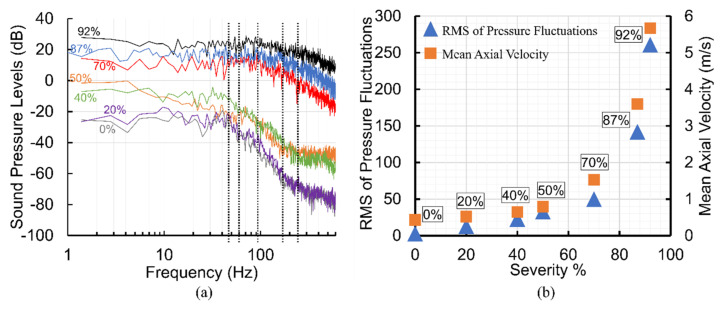
For all severity cases: (**a**) Sound pressure level (SPL) variation at different frequencies of acoustic pressure and (**b**) exponential increase in RMS of pressure fluctuations and mean axial velocity with an increase in stenosis severity.

Figure 8a shows sound pressure level (SPL) variation with the frequency of acoustic pressure fluctuations at x = 12.5 mm on the post-stenotic wall for all severity cases. This plot highlights that the frequency and energy contents of turbulent wall pressure fluctuations strongly depend on dissimilar flow structures at different stenosis severity levels. Analysis of SPL at all locations on the post-stenotic wall indicated that SPL was maximum in the fluctuating and recirculation zones, followed by the flow reattachment region. Then, the energy of wall pressure fluctuations dropped significantly after the flow became laminar. In Figure 8a, it is clear that the spectra of 0% and 20% were very similar, indicating a 20% severity does not generate significant turbulent fluctuations compared to a healthy artery. The spectral energy increased exponentially as the severity increased, shown in Figure 8b. Figure 8b illustrates the exponential increase in the $p_{RMS}^{\prime }$ at the point of maximum excitation and maximum mean axial velocity through the stenosis. The sound pressure levels of severities over 50% were significantly higher (in the order of 15–25 dB) than the lower severities.

In addition, the slopes of each spectrum changed at a specific frequency. This is known as break frequency. It is an indication of the frequency at which the flow energy, directly related to pressure fluctuations, converts into acoustic energy [9,17,46]. This transfer of energy from turbulent flow to acoustic radiation occurs when large eddies break into smaller ones with the highest fluctuations. Understanding sound characteristics such as break frequency is essential as the sound waves with higher energy, generated at break frequency, can transmit through the vessel wall and surrounding tissue and be detected at the epidermal surface utilizing non-invasive techniques. Based on the current study, each severity level had its distinct break frequency, suggesting a potential feature for the non-invasive diagnosis of stenosis at the early stages. An increase in break frequencies was observed with increased levels of severity. Break frequencies were found at about 230 Hz, 180 Hz, and 100 Hz for 92%, 87%, and 70%, respectively. For 40% and 50% severities, break frequency was determined at 55 Hz, while it was about 40 Hz for both 20% and 0% cases. In addition, the slopes of the spectrums after the break frequencies showed a decreasing trend as the severity increased. All these different characteristics observed in the measured spectra on the vessel wall with the stenosis severity progression suggest the prospective ability of the acoustic data in diagnosing and monitoring the state of stenosis.

**Figure 9:**
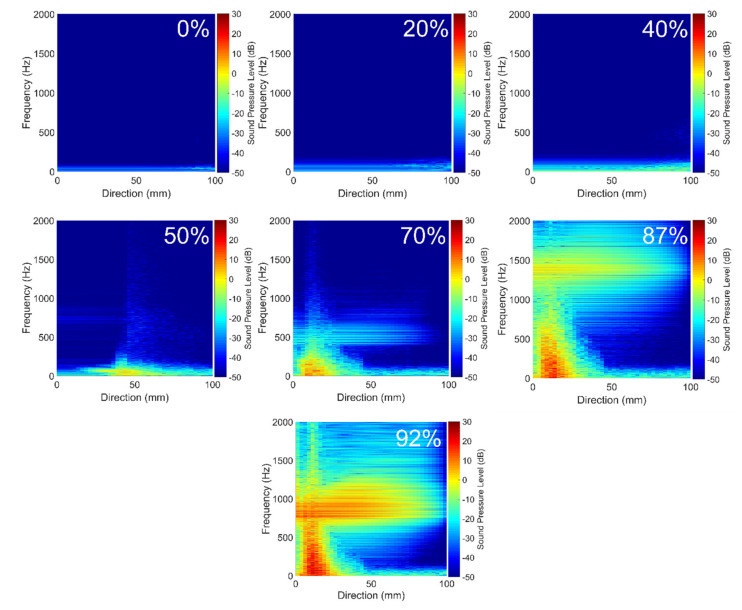
Acoustic spatial-frequency map of the post-stenotic region for all severity cases.

An acoustic spatial-frequency map of the post-stenotic region is shown in Figure 9. In healthy conditions, cardiovascular systems generate acoustic waves in a frequency range of 20–1000 Hz [17,47]. Therefore, for these unhealthy cases, the upper limit of the frequency range was set to 2 kHz to show the highest acoustic energies that existed downstream of the stenosis. It was observed that there is almost no significant peak frequency for the least severe cases. However, as the severity increased over 50%, peak frequencies with higher energies appeared in the acoustic map of the post-stenotic region. This is another indicator of the progression of the stenosis. Additionally, for high severity levels of 70%, 87%, and 92%, additional high-energy frequency ranges were observed. These ranges were approximately 400–600 Hz, 1000–1400 Hz, and 750–1000 Hz for 70%, 87%, and 92% severity, respectively. These specific additional frequencies can help us to more accurately estimate the level of stenosis.

**Figure 10:**
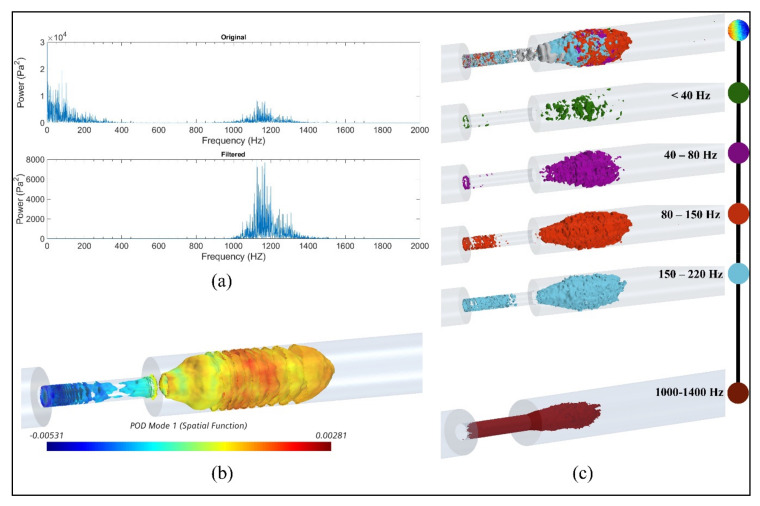
For 87% stenosis: (**a**) frequency content of the flow, (**b**) isosurfaces of proper orthogonal decomposition (POD) mode 1 of RMS of acoustic pressure, (**c**) snapshot of high-energy fluctuations for different frequency ranges and the bandwidth of 1000–1400 Hz.

The results showed that these additional high-frequency sound sources were generated inside the stenosis near the wall and existed in the shear layers where the jet core break-down happened in the post-stenotic region. As the severity increased, turbulent instabilities were initiated inside the stenosis forming relatively smaller eddies close to the wall and comparable eddies in the shear layers of the flow jet, also seen in Figure 6. The frequency content of the flow through the stenosis with 87% severity is shown, as a sample severe case, in Figure 10a. Two high-energy frequency ranges of 0–500 Hz and 1000–1400 Hz were observed. Figure 10b shows the high-energy POD mode 1 of pressure fluctuations in the flow domain for 87% severity, focused on the region of interest with the highest flow fluctuations within the artery and on the artery wall in the post-stenotic region up to 5D downstream of the stenosis. Although many studies focused on the flow in the post-stenotic region [14,15,29,33], localization of sound sources inside the stenosis has remained hardly explored. In this part, we have presented a preliminary investigation of the potential acoustic source localization inside the flow domain using spectral decomposition of flow data and POD decomposition. The flow structures shown in Figure 10 were developed from the high-intensity fluctuations through the stenosis and visualized after reconstructed from filtered pressure fluctuations. The POD technique can reduce the complexities related to time-varying hemodynamic parameters. Based on the POD mode analysis of the flow, POD mode 0, associated with mean flow, contained more than 90% of the total energy and mode 1 was determined as the next most energetic mode containing about 2.7% of the total energy. This POD mode structure showed ring-like coherent flow structures originating from the upstream edge of the stenosis. These ring-like structures were disturbed inside the stenosis due to the presence of turbulence, which can also be correlated to the length of stenosis. In addition, organized bellow-shaped flow structures were observed in the post-stenotic region within the fluctuating zone, which diminished when flow reached further downstream and became stable. These results suggested that high-energy sound sources (which may have the potential to propagate to the epidermal surface) are likely to be generated both inside the stenosis and post-stenotic region.

Although analysis of higher-order (i.e., low energy) POD modes may unveil information on important flow fluctuations with specific higher frequencies related to turbulence or geometry characteristics [39], the scope of this part limits the focus to analyze coherent structures with the highest energy fluctuations (i.e., POD mode 1) which may help identify the region(s) with high energy acoustic sources. Employing such techniques to conduct a more thorough analysis of acoustic sources to understand the origins of pressure fluctuations generated in the flow, ultimately seen in the epidermal surface for different cardiovascular diseases, is the objective of our future works.

The frequencies up to 500 Hz were also found in previous studies [15,16,29]. Therefore, the time series of $p^{\prime }$ was filtered to more specifically concentrate on the frequency range of 1000–1400 Hz to find the origin of the high-energy fluctuations in the flow. Figure 10c shows acoustic pressures using iso-surfaces of RMS of filtered pressure fluctuation for several frequency ranges (up to 220 Hz) as well as the frequency band of 1000–1400 Hz. Such visualization can help acoustic source localization of specific frequencies in the flow domain. This approach can also help characterize the coherent structures resulting from flow fluctuations at different frequency bandwidths. Similar results were observed for severities of 70% and 92% for respective frequency ranges.

This methodology can also be used for coronary arteries. The flow-induced acoustic frequencies in the stenosed coronary arteries differ from other frequencies associated with respiration, cardiac blood flow, and cardiac vibrations, which are less than ~50 Hz [29,48]. Therefore, the proposed method is feasible as a non-invasive diagnostic tool for the detection of propagated sounds from stenosed coronary arteries on the chest surface. The accuracy of the method is also determined by the sound acquisition tools with a good signal-to-noise ratio. It can be achieved with improved sensor design and advanced signal processing techniques.

We should note that the existence of a highly disturbed flow inside the stenosis and in the post-stenotic region was illustrated in this study. Turbulence significantly affects the pressure drop and wall shear stresses, leading to adverse conditions in the cardiovascular system, more specifically on the endothelial tissue of artery walls due to shear stresses and pressure fluctuations close to the wall.

## 4. Conclusions

A detailed investigation was performed in this study to unveil sound characteristics associated with stenosis progression at different severity levels. This was accomplished using computational modeling of the stenosis followed by proper orthogonal decomposition and frequency-based temporal filtering techniques to visualize the acoustic spatial-frequency map of pressure fluctuations and coherent flow structures at distinct frequency bands. The results helped localize the high-frequency turbulent pressure fluctuations in the stenosis and post-stenotic regions, focusing on acoustic fluctuations at the internal vessel wall. Comprehending these flow-induced mechanisms of the sounds propagated from stenosis can lead us to predictive techniques for diagnosing atherosclerosis before it progresses to severe cases. The findings of the current study can be summarized as follows:

- For the least severe cases, the flow solution analysis showed slight disturbances to the flow up to 50% severity. For higher severities, it was observed that the flow velocity increased significantly inside the stenosis, became unstable close to the stenosis wall, and caused significant pressure fluctuations at the plaque surface. It indicates the possibility of higher excitation of the vessel wall in the constricted flow area.
- For the most severe cases (70%, 87%, and 92%), the shear layers around the flow jet became unstable at about x = 1D, breaking into smaller eddies. The fluctuating zone was determined between 1D and 4D downstream of the stenosis, in which as the mean axial velocity decreased, the flow fluctuations increased with distance. This region contained the highest level of flow fluctuations and sound sources.
- While frequency content analysis of RMS of wall pressure fluctuations showed that the severity of 20% did not generate significant turbulent fluctuations compared to the healthy artery, the acoustic energy spectrum increased exponentially with severity levels at the point of maximum excitation at x = 2D for the most severe cases.
- Break frequencies, ranging from 40 to 230 Hz, associated with each specific severity level, were found in this study. An increase in break frequencies was also observed with increased levels of severity. These can suggest a non-invasive approach for predicting the severity of the stenosis.
- As the severity increased over 50%, peak frequencies with higher energies appeared in the acoustic spatial-frequency map of the post-stenotic region. This is another indicator of the progression of the stenosis. Furthermore, additional high-energy frequency ranges of approximately 400–600 Hz, 1000–1400 Hz, and 750–1000 Hz for 70%, 87%, and 92% severities, respectively, were observed, which can help us to estimate the level of severity at late stages.
- Visualization of acoustic pressures filtered at high frequencies of 1000–1400 Hz helped localize the source of the high-frequency fluctuations. As the severity increased, turbulent instabilities were initiated inside the stenosis forming relatively smaller eddies close to the wall and comparable eddies in the shear layers of the flow jet.

## 5. Future Works

Finding comprehensive correlations between the sound sources and variables such as different types of stenosis and flow conditions requires further detailed investigations. In the current study, we provided complementary information on the concentration of the flow fluctuations, acoustic pressure distribution on the vessel wall, and sound characteristics related to arterial stenosis progression. In future studies, additional variables (such as the stenosis profile, length of the stenosis, eccentricity,) will be investigated to find the characteristic sounds to suggest a general non-invasive and predictive method for early detection of stenosis.

The arterial wall was assumed to be rigid. The rigidity of the artery wall was based on the suggestion in previous studies. It was concluded that the wall deformation was negligible in comparable experiments and more realistic computational models at about the Reynolds number used in this study. Salman et al. found that the wall of a stenosed artery only deformed slightly due to significantly higher stiffness of the stenosis compared to the artery and surrounding tissue [14,15]. They showed that the mean flow velocities and dynamic acoustic pressures were similar for the rigid and elastic wall models. In an experimental study, Borisyuk stated that the RMS of wall pressure and the location of maximum excitation on the wall in the post-stenotic region were very close for rigid and elastic pipes [49]. It was noted that the variation in the amplitude of the RMS of wall pressure for elastic and rigid pipes became noticeable for Reynolds numbers higher than 9000. In addition, Mamun et al. also showed that the results of the flow velocity profiles at peak systole through a stenosed artery with elastic or rigid walls are similar in the upstream, throat, and downstream regions [50]. This assumption was also found in many recent studies [9,29,51,52,53,54]. We should agree that for the studies focused more on the correlation of hemodynamic parameters with the gradual development of stenosis size and the interactions between the flow and the artery wall, especially with different stiffness of stenosis, artery and surrounding tissue, the modeling assumption of an elastic wall becomes more relevant and acceptable.

Here is additional information on the objectives and improvements in our computational modeling in future studies:

- Sound analysis of flow-induced acoustics in patient-specific models derived from medical imaging, with more realistic flow properties, which may lead to specific alterations in the generated sounds compared to simplified models.
- Additional severities to derive a general correlation between the emerged signals and severity levels, which can assist to develop an algorithm for early detection of the stenosis.
- Expanding the current methodology to combine computational fluid dynamics, finite element analysis, and sound analysis techniques to conduct an in-depth investigation on the propagation of flow-induced sound waves through artery wall and the surrounding tissue.
- Combination of POD and frequency-based flow decomposition methods to study possible characteristic frequencies of flow structures for aortic aneurysm.
- Considering Pulsatile flow to account for pressure fluctuations, especially in the accelerating and decelerating phases.
- The proposed approach was performed on a few levels of severity. This needs to be tested on several cases, cross-validated by experimental sound analysis, and expanded by signal processing techniques.

It should be noted that with the use of state-of-the-art sensor technology, advanced computational modeling, and signal processing techniques, it is possible to isolate the flow-induced sounds generated in a constricted vessel from other sources of sounds originating within the body.

## Data Availability

The data presented in this study are available on request from the corresponding authors. The data are not publicly available since we are in the technology development and funding proposal preparation phases of our research project.
